# Mitochondria‐associated membranes contribution to exercise‐mediated alleviation of hepatic insulin resistance: Contrasting high‐intensity interval training with moderate‐intensity continuous training in a high‐fat diet mouse model

**DOI:** 10.1111/1753-0407.13540

**Published:** 2024-04-10

**Authors:** Xi Li, Jun Yang Yang, Wen Zhi Hu, YuXin Ruan, Hong Ying Chen, Qiang Zhang, Zhe Zhang, Zhe Shu Ding

**Affiliations:** ^1^ Key Laboratory of Adolescent Health Assessment and Exercise Intervention of Ministry of Education East China Normal University Shanghai China; ^2^ College of Physical Education & Health East China Normal University Shanghai China

**Keywords:** exercise, hepatic insulin resistance, high‐intensity interval training, mitochondria‐associated membrane, moderate‐intensity continuous training

## Abstract

**Objective:**

Mitochondria‐associated membranes (MAMs) serve pivotal functions in hepatic insulin resistance (IR). Our aim was to explore the potential role of MAMs in mitigating hepatic IR through exercise and to compare the effects of different intensities of exercise on hepatic MAMs formation in high‐fat diet (HFD) mice.

**Methods:**

Male C57BL/6J mice were fed an HFD and randomly assigned to undergo supervised high‐intensity interval training (HIIT) or moderate‐intensity continuous training (MICT). IR was evaluated using the serum triglyceride/high‐density lipoprotein cholesterol ratio (TG/HDL‐C), glucose tolerance test (GTT), and insulin tolerance test (ITT). Hepatic steatosis was observed using hematoxylin–eosin (H&E) and oil red O staining. The phosphatidylinositol 3‐kinase/protein kinase B/glycogen synthase kinase 3 beta (PI3K‐AKT‐GSK3β) signaling pathway was assessed to determine hepatic IR. MAMs were evaluated through immunofluorescence (colocalization of voltage‐dependent anion‐selective channel 1 [VDAC1] and inositol 1,4,5‐triphosphate receptor [IP3R]).

**Results:**

After 8 weeks on an HFD, there was notable inhibition of the hepatic PI3K/Akt/GSK3β signaling pathway, accompanied by a marked reduction in hepatic IP3R‐VDAC1 colocalization levels. Both 8‐week HIIT and MICT significantly enhanced the hepatic PI3K/Akt/GSK3β signaling and colocalization levels of IP3R‐VDAC1 in HFD mice, with MICT exhibiting a stronger effect on hepatic MAMs formation. Furthermore, the colocalization of hepatic IP3R‐VDAC1 positively correlated with the expression levels of phosphorylation of protein kinase B (p‐AKT) and phosphorylation of glycogen synthase kinase 3 beta (p‐GSK3β), while displaying a negative correlation with serum triglyceride/high‐density lipoprotein cholesterol levels.

**Conclusion:**

The reduction in hepatic MAMs formation induced by HFD correlates with the development of hepatic IR. Both HIIT and MICT effectively bolster hepatic MAMs formation in HFD mice, with MICT demonstrating superior efficacy. Thus, MAMs might wield a pivotal role in exercise‐induced alleviation of hepatic IR.

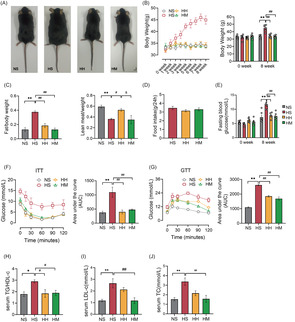

## Graphical abstract

Highlights

- The reduction of hepatic mitochondria‐associated membranes (MAMs) induced by high‐fat diet (HFD) is related to the development of hepatic insulin resistance.
- Both high‐intensity interval training (HIIT) and moderate‐intensity continuous training (MICT) effectively reverse the reduction of hepatic MAMs induced by HFD.
- MICT is more beneficial in increasing the formation of hepatic MAMs in HFD mice compared to HIIT.

## 1 INTRODUCTION

Hepatic insulin resistance (IR) is typically defined as a decreased sensitivity or responsiveness of the liver to the metabolic action of insulin.^1^ In hepatocytes, insulin binds to its receptors, activating the downstream insulin signaling pathway (phosphatidylinositol 3‐kinase/protein kinase B [PI3K/AKT]), regulating glucose utilization, and promoting glycogen storage.^2^, ^3^ Disruption of hepatic insulin signaling leads to glucose intolerance, increased gluconeogenesis, and hepatic steatosis.^1^, ^4^ This disruption further contributes to hyperglycemia and hyperlipidemia, promoting systemic IR.^5^, ^6^

Mitochondria‐associated membranes (MAMs) are dynamic domains facilitating communication between mitochondria and endoplasmic reticulum (ER).^7^ They play a crucial role in regulating various physiological processes, including mitochondrial quality control, ER stress (ERS),^8^ Ca^2+^ homeostasis, inflammation, and apoptosis.^9^ Changes in the structure and function of MAMs are linked to the occurrence and development of various diseases.^10^, ^11^, ^12^ Recent studies suggest that MAMs are involved in the control of hepatic insulin signaling, and dysregulated MAMs formation is associated with the development of hepatic IR, but the reported results are contradictory.^10^, ^13^ Nonetheless, MAMs are now presented as a subcellular structure crucial for hepatic metabolism, and modulating MAMs may represent a promising approach to prevent hepatic IR.

Exercise proves to be an effective means of preventing and alleviating hepatic IR.^14^ Numerous studies suggest that exercise enhances the function of mitochondria and ER, thereby correcting hepatic IR in high‐fat diet (HFD) mice.^15^, ^16^ It remains unclear whether mitochondrion‐ER communication is involved in this process. Furthermore, different intensities of exercise training, such as moderate‐intensity continuous training (MICT) and high‐intensity interval training (HIIT), have all been found to effectively alleviate hepatic IR. These beneficial effects may possibly occur through different mechanisms.^17^, ^18^ Currently, it is unknown whether these differences involve the regulation of hepatic MAMs.

Therefore, the purpose of this study was to verify the relationship between the hepatic MAMs formation and hepatic IR, examine whether MAMs are involved in exercise alleviating hepatic IR, and explore the difference effects of MICT and HIIT on regulating hepatic MAMs formation in HFD mice. We discovered a close association between the reduced formation of hepatic MAMs induced by HFD and the development of hepatic IR. Both HIIT and MICT effectively alleviated the reduced formation of hepatic MAMs and hepatic IR induced by HFD, with MICT showing a superior effect on MAMs formation. Our results suggest that targeting energy‐sensitive MAMs may be a therapeutic route for exercise to attenuate hepatic IR.

## 2 MATERIALS AND METHODS

### 2.1 Animals and diet intervention

Twenty‐four male C57BL/6J mice (age, 3 months) were provided by the Experimental Animal Care and Use Committee of the East China Normal University. All mice were housed in cages with a 12:12 h light–dark cycle under conventional laboratory conditions. Water and food were provided ad libitum. Following a 7‐day adaptation period, all mice from various cages underwent weighing and were marked with ear tags. Then they were randomly divided into four groups according to the principle of no significant difference in body weight: NS group (normal diet+ sedentary lifestyle, *n* = 6), HS group (high fat diet+ sedentary lifestyle, *n* = 6), HH group (high‐fat diet +HIIT, *n* = 6), and HM group (high‐fat diet +MICT, *n* = 6). Mice in NS group were fed a normal diet (Double Lion Experimental Animal Feed Technology Co., Ltd, Suzhou, China) and mice in the remaining groups were fed an HFD containing 45 kcal% fat (#D12451, research diets, Brogaarden). The weekly body weight and food intake were recorded from group‐housed mice (six mice per cage), and the daily food intake was calculated.

### 2.2 Exercise protocol

Before the formal training commenced, the training group underwent a 3‐day acclimatization exercise routine (speed: 13 m/min, duration: 20 min/day). The exercise intensity for the HH group was then determined based on this initial exercise capacity test. The running test began at a speed of 8 m/min and escalated the treadmill speed by 1 m/min every 2 min until exhaustion.^19^ Exhaustion was determined following the guidelines set by prior studies.^20^ The speed at exhaustion (Smax) was recorded, and 85% of this speed was utilized as the high‐intensity speed for the first week of training in the HH group. The low‐intensity speed was set at 40% of the high‐intensity speed. The HM group set the running speed according to the principle of having identical total running distance between groups. Before and after each training session, mice in the HM and HH groups performed standard warmup and cooldown exercises at a speed of 8 m/min for 5 min. To mitigate the impact of other environmental factors on the outcomes, mice in the NS and HS groups were exposed to the same experimental environment during the exercise regimen, excluding treadmill exercise. The training lasted 8 weeks with five times per week. The detailed protocol for exercise training is given in Table 1.

**TABLE 1:** Exercise training protocol.

Weeks	HIIT		MICT	Total running distance/m
	High‐intensity speed (2 min)	Low‐intensity speed (2 min)	Moderate intensity (60 min)	
	Repeat 12 times			
1	22 m/min	8.8 m/min	12.32 m/min	739.2
2	23 m/min	9.2 m/min	12.88 m/min	772.8
3	24 m/min	9.6 m/min	13.44 m/min	806.4
4	25 m/min	10.0 m/min	14.00 m/min	840
5	26 m/min	10.4 m/min	14.56 m/min	873.6
6	27 m/min	10.8 m/min	15.12 m/min	907.2
7	28 m/min	11.2 m/min	15.68 m/min	940.8
8	29 m/min	11.6 m/min	16.24 m/min	974.4

Abbreviations: HIIT, high‐intensity interval training; MICT, moderate‐intensity continuous training.

### 2.3 Fasting blood glucose

Fasting blood glucose (FBG) was evaluated before and after 8‐weeks of diet and exercise intervention (after a 6‐h fast, blood samples were obtained by cutting off the tip of the tail and FBG was measured using a OneTouch Ultra 2 [Lifescan, Johnson & Johnson] glucometer).

### 2.4 Body compositions

One day after the last training session, the body compositions of live mice were measured using the AccuFat MRI system (AccuFat‐1050, MAG‐MED) without anesthesia.

### 2.5 Glucose and insulin tolerance test

One week before the end of training, a glucose tolerance test (GTT) was carried out. After 6 h of fasting (8:00 a.m–2:00 p.m.), four mice in each group were randomly selected and injected intraperitoneally with d‐glucose solution diluted in 0.9% saline (1.5 g/kg b.w.i.p, Sigma‐Aldrich, USA). Blood was collected from the tails for glucose measurement at the time of 0, 15, 30, 60, 90, and 120 min.

The insulin tolerance test (ITT) was performed after 7 days of GTT. After 4 h of fasting (9:00 a.m–1:00 p.m.), four mice in each group that had completed GTT were injected intraperitoneally with insulin diluted in 0.9% saline (0.5 U/kg b.w. i.p, Novolin R, Novo Nordisk). Blood was collected from the tails for glucose measurement at the time of 0, 15, 30, 60, 90, and 120 min. The areas under the curve (AUC) were calculated by the GraphPad Prism software.^21^

Three days later, mice were fasted overnight and serum was sampled by extracting the eyeball blood after mice were anesthetized with isoflurane. Liver tissues were harvested for further analysis. Liver histology samples were obtained from the same cohort of mice (*n* = 3) utilized in GTT and ITT experiments. The sampling process was carried out by personnel blinded to the animal grouping. Subsequently, this set of samples was embedded in either paraffin or optimal cutting temperature and utilized for hematoxylin–eosin (H&E) staining, Oil Red O staining, and immunofluorescence experiments.

### 2.6 Blood parameters

The concentrations of serum triglyceride (TG), total cholesterol (TC), high‐density lipoprotein cholesterol (HDL‐C), and low‐density lipoprotein cholesterol (LDL‐C) were determined by using the biochemistry kits purchased from Nanjing Jiancheng Bioengineering Institute (Nanjing, China).Then, the serum TG/HDL‐C ratio was calculated as an index to evaluate IR.^22^, ^23^

### 2.7 Liver histology

These liver samples were immersed in 10% neutral buffered formalin for 24 h and dehydrated using 70% ethanol. Liver tissue blocks were embedded in the paraffin and cut into 8 μm thick sections for H&E. The same liver lobe of each animal was captured by using the Nikon View software (Nikon DS‐U3, Japan).^24^

For the Oil Red O staining, liver tissues from mice were embedded in Tissue‐Tek OCT (Sakura Finetek, Torrance, CA, USA) in a cryostat mold and cut into 5 μm sections. Analysis was performed by an observer blinded to the treatment groups. lipid content in Oil Red O sections were semiquantified using Image‐Pro Plus analysis software program (Image‐Pro Plus 6.0; Media Cybernetics, Washington, DC, USA).

### 2.8 Immunofluorescence staining and quantification

The quantification for immunofluorescence staining referred to previous studies. Briefly, liver tissues were embedded in paraffin and sectioned into 10 μm thick sections, deparaffinized and rehydrated, followed by heat‐induced epitope retrieval.^25^ Sections were then blocked with BSA and incubated overnight at 4°C with primary antibody to inositol 1,4,5‐triphosphate receptor (IP3R; GB11742, Servicebio, Wuhan, China). After three times washing with PBS, corresponding HRP‐labeled secondary antibody was added and incubated at room temperature for 50 min. The sections were then added FITC (Servicebio, Wuhan) and incubated in dark for 10 min, followed by antigen retrieval with EDTA antigen retrieval buffer (1 mM EDTA, pH 8.0). Subsequently, these sections were incubated with the second primary antibody voltage‐dependent anion‐selective channel 1 (VDAC1; GB11251, Servicebio, Wuhan, China), then added corresponding HRP‐labeled secondary antibody and incubated for 50 min at room temperature in the dark. Liver tissue samples from three mice in each experimental group were employed for sectioning and staining. For each stained section, three random fields of view were selected for observation and photography. (A total of nine fields of view per group were used to analyze the colocalization of IP3R‐VDAC1.) Quantitative analysis of fluorescence colocalization was performed using ImageJ Colocalization Finder plug‐in software.

### 2.9 Liver triglyceride determination

The liver tissues of mice were homogenized with absolute ethanol to prepare 10% homogenates, then centrifugated (4°C, 2500 rpm, 10 min), and the supernatant was collected. The TG content in the livers were determined using a Triglyceride Assay Kit according to the manufacturer's protocol (Nanjing Jiancheng Bioengineering Institute, China).

### 2.10 Western blot

Western blot analysis was conducted as previously described.^26^ Fresh frozen postmortem liver tissue samples were obtained from the same cohort of mice (*n* = 3) utilized in liver histology experiments. Approximately 20 mg of tissue was removed from each sample and thoroughly homogenized in RIPA buffer (0.1% sodium deoxycholate, 0.5% NP‐40, 150 mM NaCl, 50 mM Tris‐Cl, pH 7.5) supplemented with PMSF (ST506), phosphatases, and protease inhibitors (P1045). The supernatant was collected and used to measure total protein concentration with the BCA assay (BCA Protein Assay Kit, 23 227, Thermo Scientific, USA). Loading buffer and PBS were added to the protein sample to make the final concentration 5 μg/μL.

25 micrograms of protein were separated on a 10%–12% SDS‐PAGE gel by electrophoresis and then transferred to an immobilon PVDF membrane. The membrane was blocked with 5% BSA (MPbio, 0218054950, California, USA) in TBST buffer at room temperature for 1 h. Primary antibodies for the following were added to the PVDF membranes and then incubated overnight at 4°C: phospho‐PI3K (Affinity, #AF3241, 1:1000 dilution), AKT (CST, #4691, 1:1000 dilution), phospho‐AKT (Ser473) (CST, #4060, 1:2000 dilution), phospho‐glycogen synthase kinase 3 beta (GSK3β) (Ser9) (Affinity, AF2016,1:1000 dilution), Fis1 (mitochondrial fission protein 1; Proteintech, 10 956‐1‐AP, 1:1000 dilution), OPA1 (optic atrophy 1; Santa Cruz, SC‐367890, 1:200 dilution), PINK1 (PTEN induced putative kinase 1; Beyotime, AF7755, 1:1000 dilution), Parkin (E3 ubiquitin‐protein ligase parkin; CST, #32833, 1:1000 dilution), LC3B (microtubule‐associated‐proteinlight‐chain‐3B; CST, #3868, 1:1000 dilution), BNIP3 (BCL2/adenovirus E1B 19 kDa protein interacting protein 3; Beyotime, AF6330, 1:1000 dilution), PERK (protein kinase R like ER kinase; Abcam, ab229912, 1:1000 dilution), phospho‐PERK (Thr982) (Affinity, DF7576, 1:2000 dilution), phospho‐eIF2α (Ser51/52) (Affinity, AF3087, 1:1000 dilution), β‐Tublin (Affinity, AF7011, 1:2500 dilution), GAPDH (Servicebio, GB15004, 1:2000 dilution), and HSP90 (Abcam, ab178854, 1:5000 dilution) 1 × TBST containing 3% BSA. The membranes were rinsed three times in TBST, and the secondary antirabbit or antimouse IgG HRP conjugate (1:10000, Jackson) were added and incubated for 1 h at room temperature, followed by another three washes in TBST. The images of protein bands were captured and analyzed by the ChemiDoc MP Imaging System (BIORAD, California, USA). β‐Tublin, GAPDH, and HSP90 were used as loading control. All protein abbreviations can be found in Table 2.

**TABLE 2:** Abbreviations.

Abbreviation	Full name
PI3K	Phosphatidylinositol 3‐kinase
AKT	Protein Kinase B
GSK3β	Glycogen Synthase Kinase 3 Beta
VDAC1	Voltage‐dependent anion‐selective channel protein
IP3R	inositol 1,4,5‐triphate receptor
Fis1	mitochondrial fission protein 1
OPA1	optic atrophy 1
PINK1	PTEN induced putative kinase 1
Parkin	E3 ubiquitin‐protein ligase parkin
LC3	microtubule‐associated‐proteinlight‐chain‐3
BNIP3	BCL2/adenovirus E1B 19 kDa protein interacting protein 3
PERK	protein kinase R like ER kinase
eIF2α	eukaryotic translation initiation factor 2A
β‐Tublin	beta tubulin
GAPDH	glyceraldehyde‐3‐phosphate dehydrogenase
HSP90	heat shock protein 90
Mfn2	Mitofusion2

### 2.11 Statistical analysis

Statistical analysis was conducted utilizing GraphPad Prism (version 7.0, La Jolla, CA, USA). Group differences were assessed through one‐way analysis of variance (ANOVA) with Sidak post hoc testing. Repeated‐measures ANOVA was employed for body weight plots, GTT, and ITT data. The quantitative colocalization analysis of IP3R‐VDAC1 was executed using the ImageJ Colocalization Finder plug‐in software. Correlations were computed using Pearson correlation. No methods for handling missing data were applied, as there were no instances of missing data. Statistical significance was set at *p* < .05 or .01. The data were presented as mean ± SEM.

## 3 RESULTS

### 3.1 HIIT and MICT attenuated IR in HFD mice

**FIGURE 1:**
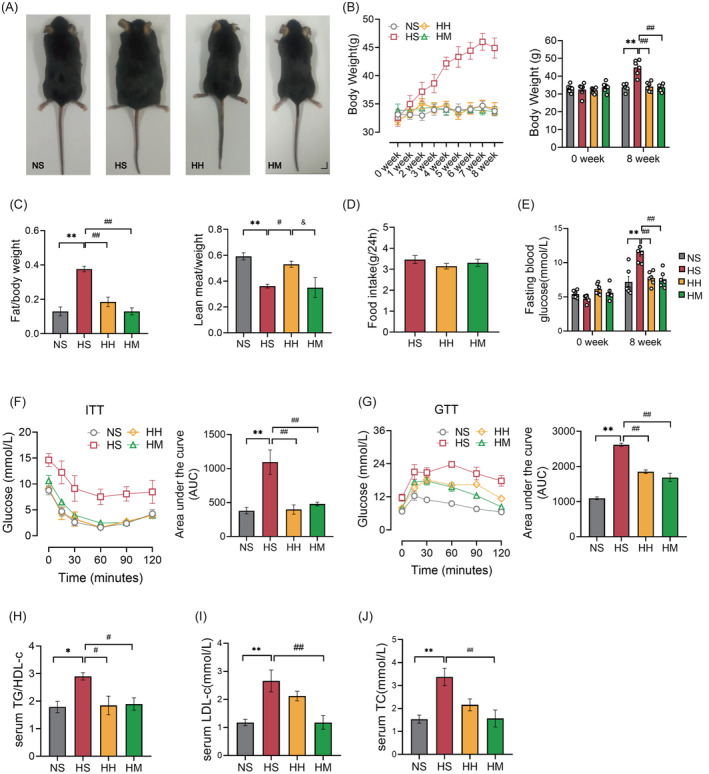
HIIT and MICT attenuated IR in HFD mice. (A) Appearance of mice in each group after 8 weeks of dietary intervention (scale bar: 10 mm). (B) Body weight. (C) Body composition. (D) Mean daily food intake of mice. (E) FBG level. (F) ITT (*n* = 4). (G) GTT (*n* = 4). (H) Serum TG/HDL‐C (*n* = 4). (I) Serum LDL‐C (*n* = 4). (J) Serum TC (*n* = 4). Unless otherwise specified, six mice per group were used in each experiment. Data are presented as means ± SEM. *p < .05, **p < .01 versus NS; #*p* < .05, ##*p* < .01 versus HS; &*p* < .05 versus HH. FBG, fasting blood glucose; GTT, glucose tolerance test; HDL‐C, high‐density lipoprotein cholesterol ratio; HFD, high fat diet; HH, high fat diet +HIIT; HIIT, high‐intensity interval training; HM, high fat diet +MICT; HS, high fat diet+ sedentary lifestyle; IR, insulin resistance; ITT, insulin tolerance test; LDL‐C, low‐density lipoprotein‐cholesterol; MICT, moderate‐intensity continuous training; NS, normal diet+ sedentary lifestyle; TC, total cholesterol; TG, triglyceride.

We conducted an 8‐week‐HFD feeding to replicate HFD‐induced IR. Upon completion of the 8‐week HFD intervention, the HS group exhibited significantly higher body size, body weight (*p* < .01), and fat mass (*p* < .01) compared to the NS group (Figure 1A–C). Conversely, the lean body mass (*p* < .01) in the HS group significantly decreased compared to that in the NS group (Figure 1C). Additionally, serum TC (*p* < .01), LDL‐C (*p* < .01), and TG/HDL‐C ratio (*p* < .05) levels in the HS group were significantly increased compared to the NS group, indicating the development of hyperlipidemia due to long‐term HFD (Figure 1H–J). Furthermore, HFD led to increased FBG (*p* < .01) and glucose intolerance (assessed through GTT) (*p* < .01) and decreased insulin sensitivity (assessed through ITT) (*p* < .01) (Figure 1E–G). These findings affirm that 8‐week HFD feeding successfully induces IR.

Subsequently, we investigated the effects of HIIT or MICT on IR in HFD mice. Following the 8‐week exercise combined with HFD intervention, the HH and HM groups demonstrated significantly lower body size and weight (*p* < .01), fat mass (*p* < .01), FBG (*p* < 0.01), and serum TG/HDL‐C ratio (*p* < .05) compared to the HS group (Figure 1A–C,E,H). Furthermore, both the HH and HM groups exhibited significant improvements in insulin sensitivity (assessed through ITT) (*p* < .01) and glucose tolerance (assessed through GTT) (*p* < .01) compared to the HS group (Figure 1F,G). Importantly, there was no significant difference in food intake (*p* > .05) between mice in the HH and HM groups and those in the HS group (Figure 1D). These results suggest that both HIIT and MICT effectively alleviate IR in HFD mice.

Interestingly, serum LDL‐C (*p* < .01) and TC levels (*p* < .01) in the HM group were significantly lower than those observed in the HS group, whereas the lean body mass remained unchanged (Figure 1C,I,J). The lean body mass (*p* < .05) in the HH group was significantly higher than that in the HS group. However, serum LDL‐C (*p* > .05) and TC (*p* > .05) levels in the HH group did not differ significantly from those in the HS group (Figure 1C,I,J). These results suggest that HIIT may promote muscle gain, whereas MICT appears to facilitate fat loss in HFD mice.

### 3.2 HIIT and MICT alleviated hepatic IR in HFD mice

**FIGURE 2:**
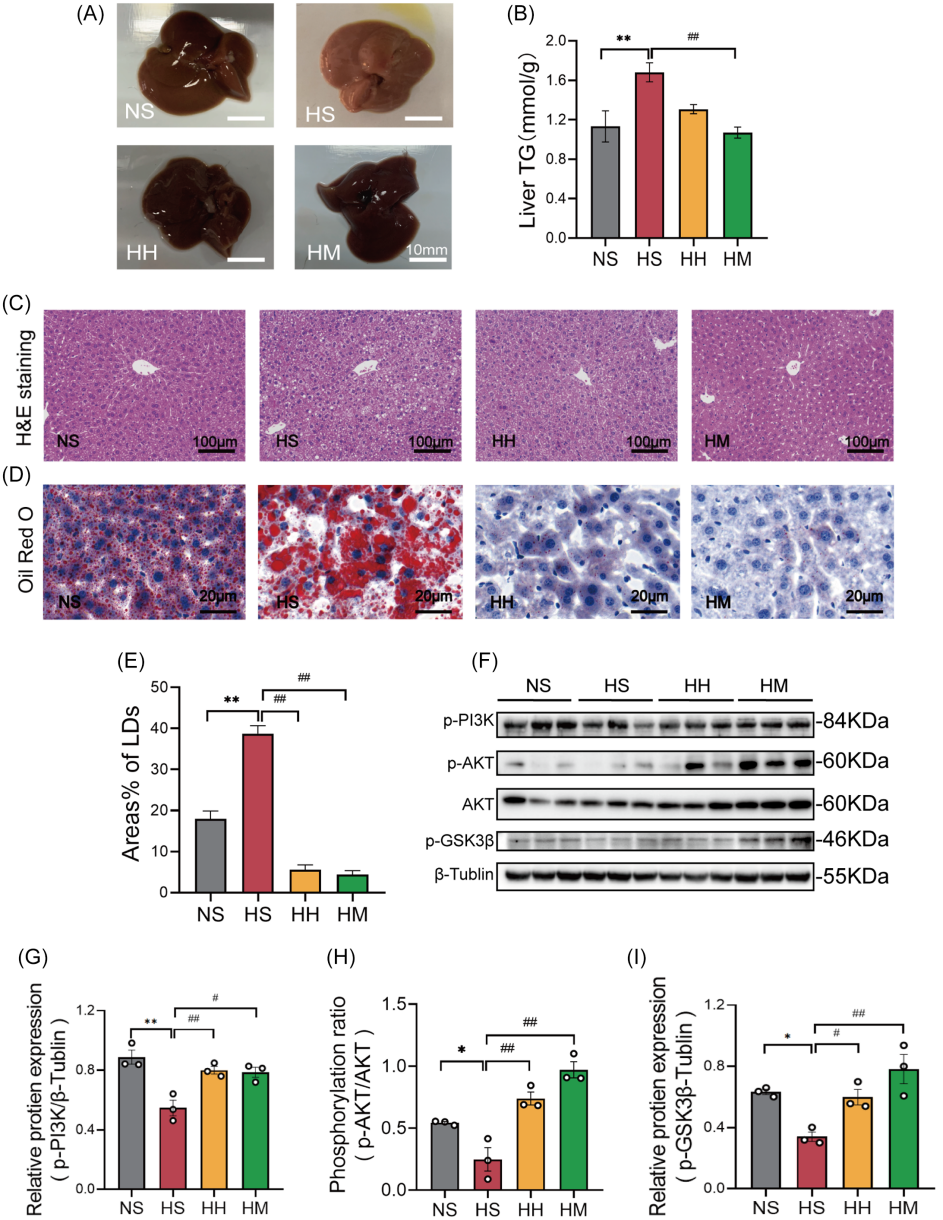
HIIT and MICT alleviated hepatic IR in HFD mice. (A) Representative macroscopic morphology (scale bar: 10 mm). (B) Liver TG (*n* = 4). (C) H&E staining (scar bar: 100 μm) (D) Oil red O staining (scar bar: 20 μm). (E) Oil red O staining analysis. (F) Representative western blot images of p‐PI3K, p‐GSK3β, AKT, and p‐AKT (G–I) quantified data of western blot images. Unless otherwise specified, three mice per group were used in each experiment. Data are presented as means ± SEM. *p < 0.05, **p < 0.01 versus NS; #*p* < 0.05, ##*p* < 0.01 versus HS. AKT, protein kinase B; H&E, hematoxylin–eosin; HFD, high fat diet; HH, high fat diet +HIIT; HIIT, high‐intensity interval training; HM, high fat diet +MICT; HS, high fat diet+ sedentary lifestyle; IR, insulin resistance; LDs, lipid droplets; MICT, moderate‐intensity continuous training; NS, normal diet+ sedentary lifestyle; p‐AKT, phosphorylation of protein kinase B; p‐GSK3β, phosphorylation of glycogen synthase kinase 3 beta; p‐PI3K, phosphorylation of phosphatidylinositol 3‐kinase; TG, triglyceride.

Observations were made on the appearance and histological structure of livers across different groups. As depicted in Figure 2A, the livers in the NS, HH, and HM groups appeared bright red, firm, and well defined. Conversely, the liver in the HS group appeared light yellow, round, and fatty, displaying typical characteristics of a fatty liver. H&E and Oil Red O staining of liver tissues were employed to assess histopathological changes and visualize lipid droplets, respectively (Figure 2C,D). Mice in the HS group exhibited hepatocyte ballooning and disorganized arrangement compared to those in the NS group. In contrast, hepatocytes in the HH and HM groups displayed a well‐maintained structure and orderly arrangement (Figure 2C). Furthermore, a considerable number of large‐sized lipid droplets were observed in the liver sections of mice in the HS group compared to those in the NS group (Figure 2D). Both the HH and HM groups showed a significant reduction in the size and area percentage of hepatocyte lipid droplets (*p* < .01) compared to the HS group (Figure 2D,E).

Interestingly, hepatic TG levels (*p* < .01) in the HM group were significantly lower than those in the HS group (Figure 2B). However, no significant difference in hepatic TG levels (*p* > .05) was observed between mice in the HH and HS groups (Figure 2B). These findings indicate that HFD feeding induced hepatic steatosis, and both HIIT and MICT effectively mitigated this condition. Nevertheless, it appears that HIIT primarily does not act by reducing hepatic TG content.

Figure 2F illustrates our further exploration of the impact of a HFD and HFD combined with exercise on the insulin signaling pathway (PI3K/AKT/GSK3β). The phosphorylation levels of PI3K (*p* < .01), AKT (*p* < .05), and GSK3β (*p* < .05) in the HS group were significantly lower than those in the NS group. However, both the HH and HM groups exhibited a significant increase in the phosphorylation levels of PI3K (*p* < .05), AKT (*p* < .01), and GSK3β (*p* < .05) compared to the HS group (Figure 2G–I). These findings indicate that HIIT and MICT effectively restore the impaired hepatic PI3K/AKT/GSK3β signaling pathway.

### 3.3 MICT was more beneficial to the repair of hepatic MAMs in HFD mice compared with HIIT

**FIGURE 3:**
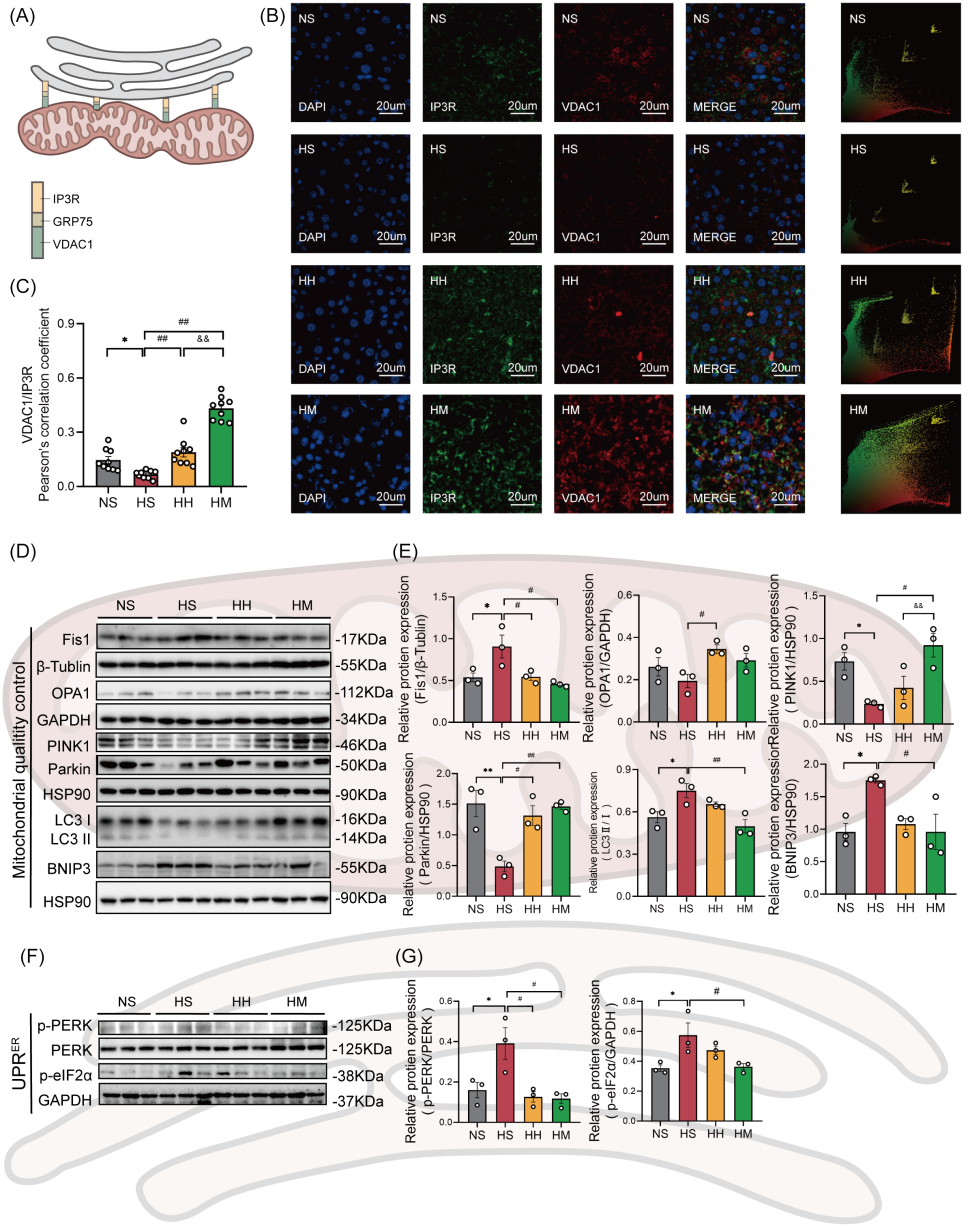
MICT was more beneficial to the repair of hepatic MAMs in HFD mice compared with HIIT. (A) model of MAMs. (B) Representative immunofluorescence images (bar: 20 μm; green: IP3R; blue: DAPI; red: VDAC1) and scatter dot plot of colocalization co‐efficiency of VDAC1 and IP3R. (C) Statistical map of VDAC1‐IP3R colocalization. (D) Representative Western blot images of Fis1, OPA1, PINK1, Parkin, LC3II/I, BNIP3. (E) quantified data of Fis1, OPA1, PINK1, Parkin, LC3II/I, BNIP3. (F) Representative western blot images of p‐PERK, PERK, p‐eIF2α. (G) quantified data of p‐PERK/PERK and p‐eIF2α. Unless otherwise specified, three mice per group were used in each experiment. Data are presented as means ± SEM (*n* = 3 per group). *p < .05, **p < .01 versus NS; #*p* < .05, ##*p* < .01 versus HS; &&*p* < .01 versus HH. BNIP3, BCL2/adenovirus E1B 19 kDa protein interacting protein 3; Fis1, mitochondrial fission protein 1; HFD, high fat diet; HH, high fat diet +HIIT; HIIT, high‐intensity interval training; HM, high fat diet +MICT; HS, high fat diet+ sedentary lifestyle; IP3R, inositol 1,4,5‐triphosphate receptor; LC3II/I, microtubule‐associated‐proteinlight‐chain‐3B; MAMs, mitochondria‐associated membranes; MICT, moderate‐intensity continuous training; NS, normal diet+ sedentary lifestyle; OPA1, optic atrophy 1; Parkin, E3 ubiquitin‐protein ligase parkin; p‐eIF2α, phosphorylation of eukaryotic translation initiation factor 2A; PERK, protein kinase R like ER kinase; PINK1, PTEN induced putative kinase 1; p‐PERK, phosphorylation of protein kinase R like ER kinase; UPR^ER^, enhanced endoplasmic reticulum unfolded protein response; VDAC1, voltage‐dependent anion‐selective channel protein.

As shown in Figure 3A, the ER outer membrane protein IP3R interacts with the mitochondrial outer membrane protein VDAC1 through GRP75 to form a tether connecting mitochondria and the ER. We evaluated the formation of MAMs by immunofluorescence colocalization of IP3R and VDAC1 (Figure 3B). The immunofluorescence quantification data are presented in Figure 3C. The colocalization levels of IP3R and VDAC1 (*p* < .05) in liver tissues of the HS group were significantly lower than those in the NS group. In comparison to the HS group, the colocalization levels of IP3R and VDAC1 (*p* < .01) in liver tissue were significantly increased in both the HH and HM groups. Interestingly, the HM group exhibited significant improvements in the colocalization levels of IP3R and VDAC1 (*p* < .01) compared to the HH group. These findings suggest that both HIIT and MICT enhance the formation of hepatic MAMs in HFD mice, with MICT demonstrating greater efficacy.

Genetic evidence showed that excessive mitochondrial fission, abnormal mitophagy, and an enhanced ER unfolded protein response (UPR^ER^) are important manifestations of MAMs damage.^8^, ^26^ In Figure 3D,F, the status of hepatic MAMs was evaluated by detecting the protein levels associated to the hepatic mitochondria fission and fusion (Fis1 and OPA1), mitophagy (PINK1, Parkin, LC3II/I, and BNIP3), and UPR^ER^ (p‐PERK [phosphorylation of protein kinase R like ER kinase] and p‐eIF2α). In comparison to the NS group, the HS group exhibited significantly increased protein levels of Fis1 (*p* < .05), LC3II/I (*p* < .05), BNIP3 (*p* < .05), p‐PERK (*p* < .05), and p‐eIF2α (*p* < .05) (Figure 3E,G). Conversely, the protein levels of PINK1 (*p* < .05) and Parkin (*p* < .01) in the HS group were notably lower than those in the NS group (Figure 3E). Although a decline in OPA1 protein levels was observed in the HS group compared to the NS group, this difference did not reach statistical significance (Figure 3E). Notably, the HH and HM groups exhibited significant decreases in the protein levels of Fis1 (*p* < .05) and p‐PERK (*p* < .05), alongside a significant increase in Parkin protein level (*p* < .05) compared with the HS group (Figure 3E,G).

The HH group displayed significantly higher levels of OPA1 (*p* < .05) compared to the HS group. However, the protein levels of PINK1, LC3 II/I, and p‐eIF2α remained unchanged (Figure 3E,G). Furthermore, a decreasing trend in BNIP3 protein levels was observed in the HH group compared to the NS group, but statistical significance was not reached (*p* = .06) (Figure 3E). In contrast, the HM group showed a significant increase in PINK1 protein levels (*p* < .05) and significant decreases in LC3 II/I (*p* < .01), BNIP3 (*p* < .05), and p‐eIF2α (*p* < .05) compared with the HS group (Figure 3E,G). In addition, there was no significant difference in OPA1 expression between the HM and HS groups (Figure 3E).

### 3.4 Hepatic MAMs formation was negatively related to hepatic IR

**FIGURE 4:**
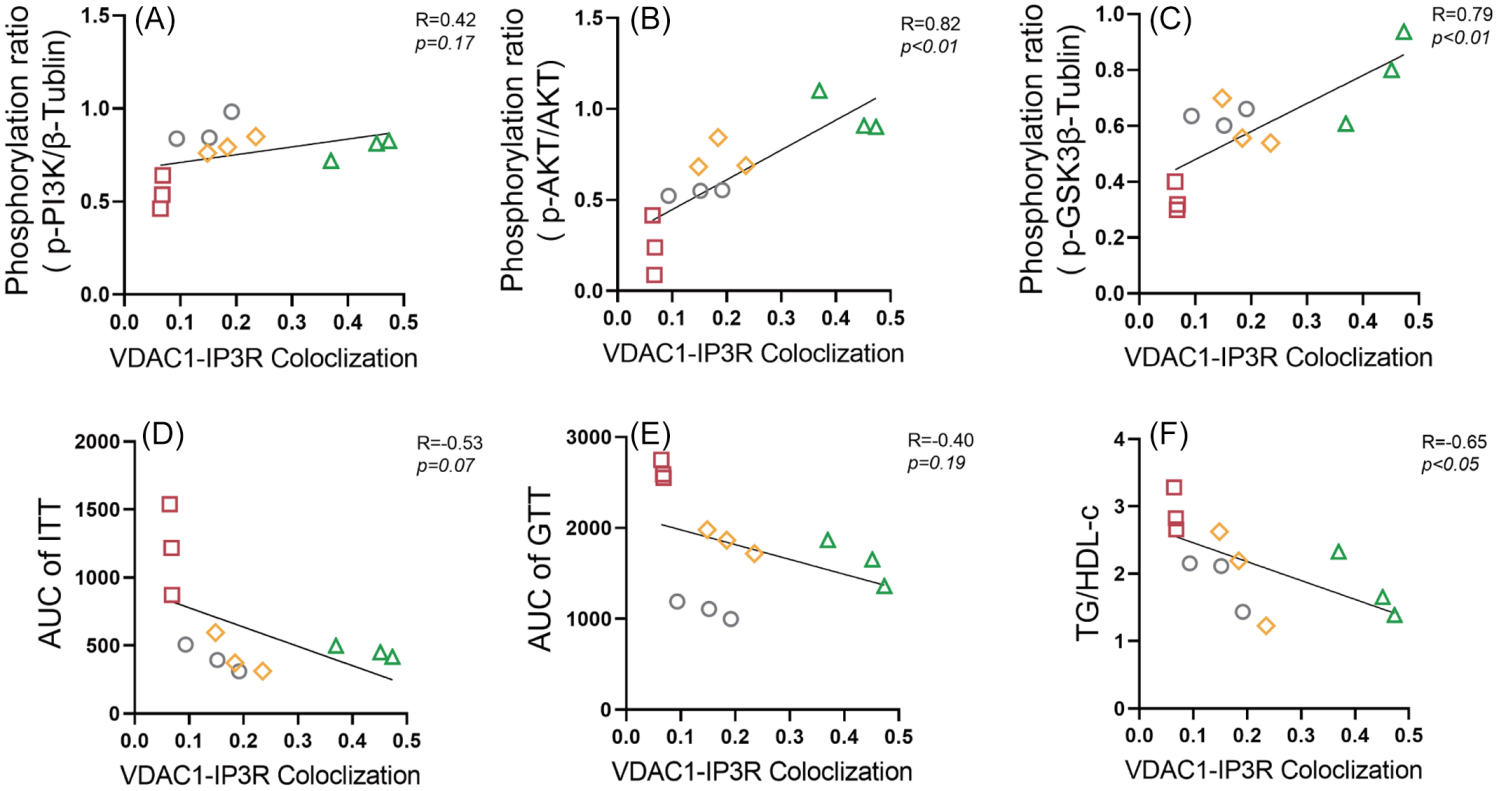
Hepatic MAMs formation was negatively related to hepatic IR. (A) Correlation of hepatic VDAC1‐IP3R interaction with p‐PI3K. (B) Correlation of hepatic VDAC1‐IP3R interaction with p‐AKT/AKT. (C) Correlation of hepatic VDAC1‐IP3R interaction with p‐GSK3β. (D) Correlation of hepatic VDAC1‐IP3R interaction with ITT AUC. (E) Correlation of hepatic VDAC1‐IP3R interaction with GTT AUC. (F) Correlation of hepatic VDAC1‐IP3R interaction with serum TG/HDL‐C. Unless otherwise specified, three mice per group were used in each experiment. Data are presented as means ± SEM. AKT, protein kinase B; AUC, areas under the curve; GTT, glucose tolerance test; HFD, high fat diet; HDL‐C, high‐density lipoprotein cholesterol ratio; IP3R, inositol 1,4,5‐triphate receptor; ITT, insulin tolerance test; MAMs, mitochondria‐associated membranes; p‐AKT: phosphorylation of protein kinase B; p‐GSK3β, phosphorylation of glycogen synthase kinase 3 Beta; p‐PI3K, phosphorylation of phosphatidylinositol 3‐kinase; TG, triglyceride; VDAC1, voltage‐dependent anion‐selective channel protein.

To investigate the relationship between hepatic MAMs and hepatic IR, we analyzed the correlation between protein levels of key molecules in the hepatic insulin signaling pathway (PI3K/AKT/GSK3β) and the degree of IP3R‐VDAC1 colocalization. The colocalization of IP3R‐VDAC1 showed a significant positive correlation with the protein levels of p‐AKT (*R* = 0.82, *p* < .01; Figure 4B) and phosphorylation of glycogen synthase kinase 3 beta (p‐GSK3β; *R* = 0.79, *p* < .01; Figure 4C). However, no significant correlation was observed between the colocalization of IP3R‐VDAC1 and the protein level of phosphorylation of PI3K (p‐PI3K; *R* = 0.42, *p* = .17; Figure 4A).

We further explore the relationship between hepatic MAMs and IR. Our findings indicate a notable negative correlation between the colocalization of IP3R‐VDAC1 and serum TG/HDL‐C (*R* = −0.65, *p* < .05, Figure 4F). Nevertheless, no significant correlation emerged between the colocalization of IP3R‐VDAC1 and either the AUC of ITT (*R* = −0.53, *p* = .07, Figure 4D) or the AUC of GTT (*R* = −0.40, *p* = .19, Figure 4E).

## 4 DISCUSSION

### 4.1 The decrease in hepatic MAMs induced by HFD correlates with the development of hepatic IR

In our study, we established that an 8‐week 45% HFD was sufficient to induce hepatic IR. This was determined by evaluating hepatic steatosis severity and the phosphorylation levels of key proteins in the hepatic insulin signaling pathway (PI3K/AKT/GSK3β). Hepatic IR triggers uncontrolled hepatic gluconeogenesis,^27^ leading to elevated blood glucose levels and consequently contributing to systemic IR.^28^ Consistently, we also observed systemic IR phenotypes in HFD mice, including increased blood glucose, elevated blood lipids, impaired glucose tolerance, and diminished insulin sensitivity.

MAMs pivotal in regulating hepatic insulin signal transduction.^11^, ^29^ Hence, we evaluated MAMs formation by employing double immunofluorescent labeling of tethering proteins IP3R and VDAC1, situated on the ER and mitochondrial membranes, respectively. We observed a significant decrease in the colocalization of IP3R and VDAC1 in the liver following 8 weeks of 45% HFD treatment.

Additionally, blocking the formation of MAMs leads to mitochondrial fragmentation, inhibition of mitophagy, and aggravated ERS.^8^, ^30^ Consequently, we analyzed the expression of proteins associated with hepatic mitochondrial fission/fusion, mitophagy, and ERS. As expected, the HFD not only triggered excessive division of hepatic mitochondria but also suppressed mitophagy, leading to a substantial increase in ERS. This evidence supports the conclusion that an HFD induces a decline in hepatic MAMs formation. Consistent with our findings, a reduction in hepatic MAMs formation was observed in 16‐week and 12‐week‐old OB/OB mice fed a 45% high‐fat high‐sugar diet.^31^ Despite indications of a connection between dysregulated hepatic MAMs formation and the occurrence and development of hepatic IR, conflicting data still exist.^10^, ^13^

Our investigation unveiled a positive correlation between the extent of VDAC1‐IP3R colocalization in the liver and the levels of phosphorylated AKT (p‐AKT) and phosphorylated GSK3β (p‐GSK3β) proteins. Simultaneously, it demonstrated a negative correlation with the serum TG/HDL‐C ratio. These findings imply that the decline in hepatic MAMs induced by an HFD is linked to the development of hepatic IR.

### 4.2 HIIT and MICT enhance hepatic MAMs formation and alleviate hepatic IR in HFD mice

We have previously established that the decrease in hepatic MAMs formation induced by HFD is associated with the development of hepatic IR. Regular exercise training has been proven to alleviate obesity, metabolic abnormalities, and IR induced by HFD.^32^, ^33^ However, the role of MAMs in exercise‐mediated alleviation of hepatic IR remains unclear. In a mouse model of type 2 diabetes mellitus, we previously observed a notable increase in the expression of the skeletal muscle MAMs‐related protein Sigma‐1R after exercise, concomitant with a reduction in skeletal muscle IR.^34^ The expression of Mfn2 has also been linked to MAMs formation, as knocking out or silencing Mfn2 leads to the dissociation of the ER and mitochondria.^35^ Liver‐specific knockout of Mfn2 not only causes excessive mitochondrial fission but also inhibits insulin signaling in liver tissue, inducing susceptibility to IR.^36^ Long‐term aerobic training significantly upregulated the expression of Mfn2.^37^ However, HIIT and MICT have significant differences in their effects on Mfn2 expression levels in skeletal muscle.^38^ The evidence herein suggests that exercise likely plays a role in regulating the formation of MAMs, which could be a potential mechanism through which exercise alleviates tissue IR. However, there may be variations in the regulation of MAMs formation by different intensities of exercise training.

Therefore, we respectively employed two exercise intervention modes, HIIT and MICT, to investigate the impact of exercise on hepatic MAMs in mice subjected to an HFD. Both HIIT and MICT have been established as beneficial for alleviating hepatic IR. Our findings showed that both MICT and HIIT effectively augmented the colocalization of IP3R and VDAC1 in the livers of HFD mice.

In yeast cells, the uncoupling of the ER‐mitochondria encounter structure (ERMES/MAMs) diminishes mitophagy, whereas the artificial restoration of ERMES reinstates mitophagy.^39^, ^40^ Indeed, in both mammals and yeast, MAMs/ERMES regulate the selective degradation of unused or damaged mitochondria.^41^ Furthermore, a reduction in the expression of the mitophagy‐related protein PINK1 was observed in mice fed an HFD, whereas overexpression of PINK1 enhanced glucose uptake and downregulate gluconeogenic enzyme levels.^42^ This suggests that the regulation of mitophagy levels is influenced by MAMs. Moreover, the induction of IR by HFD appears to be associated with a decrease in mitophagy levels.

In this study, we observed that MICT reversed the HFD‐induced decrease in the expression of mitophagy proteins PINK1 and Parkin, and downregulated the expression levels of LC3I/II and BNIP3. On the other hand, HIIT only upregulated the expression of Parkin in HFD mice. Additionally, disruption of MAMs formation leads to excessive mitochondrial fragmentation and heightened ERS.^8^, ^43^ ERS is considered a risk factor for IR,^44^ and excessive mitochondrial fission is a significant molecular characteristic in diabetic mice.^45^ This implies that the excessive mitochondrial fragmentation and ERS resulting from MAMs damage may also be associated with the development of IR. We observed that both HIIT and MICT significantly downregulated the expression levels of the mitochondrial fission protein, Fis1, and the ERS‐related protein, p‐PERK. Additionally, MICT exhibited a notable reduction in the expression level of p‐eIF2α, another signature protein associated with ERS. Importantly, both MICT and HIIT effectively mitigated hepatic IR induced by an HFD and the subsequent systemic IR.

Our findings suggest that both MICT and HIIT reverse the HFD‐induced decrease in hepatic MAMs formation. This reversal could be a crucial factor in alleviating hepatic IR and subsequent systemic IR. Nevertheless, it is essential to note that further experimental validation is necessary to consolidate these findings.

### 4.3 In comparison to HIIT, MICT more effectively improves hepatic MAMs formation and systemic lipid accumulation in HFD mice

Although both HIIT and MICT demonstrated a significant promotion of MAMs formation in the liver of HFD mice, we made the surprising observation that MICT was more effective in this regard compared to HIIT. This was primarily manifested by the enhanced colocalization of IP3R and VDAC1 in the liver of HFD mice subjected to MICT, accompanied by an increase in the expression of mitophagy proteins and a decrease in the expression of ERS‐related proteins. As previously mentioned, different intensities of exercise training exhibit variations in the regulation of the expression levels of molecules associated with MAMs formation,^38^ making our observed outcomes less surprising. What adds an interesting dimension is the simultaneous observation of certain metabolic phenotypic differences under these two exercise interventions. For instance, although MICT and HIIT exhibited similar capabilities in reducing body weight, FBG levels, and improving glucose tolerance and insulin sensitivity, MICT demonstrated superior effects in regulating lipid metabolism in HFD mice. This was evidenced by reductions in body fat mass, serum LDL‐C and TC levels, as well as hepatic TG content. Hence, we hypothesize that lipid signals from both intracellular and extracellular sources may serve as critical regulatory factors for MAMs formation. Unfortunately, the potential mechanisms underlying these effects were not explored in this study.

In summary, our study establishes a correlation between the reduction in hepatic MAMs formation induced by HFD and the development of hepatic IR. Both HIIT and MICT prove effective in rescuing the decreased hepatic MAMs formation induced by HFD. Furthermore, the impact of MICT on hepatic MAMs formation appears to be more pronounced compared to HIIT, potentially linked to alterations in lipid metabolism. Although our findings indeed confirm that exercise enhances hepatic MAMs formation in HFD mice and concurrently alleviates hepatic IR, further research is warranted to thoroughly investigate the underlying mechanisms responsible for these effects.

## AUTHOR CONTRIBUTIONS

Xi Li, Jun Yang Yang, Zhe Zhang, and Zhe Shu Ding conceived completely the project. Xi Li and Jun Yang Yang performed experiments and wrote the manuscript. Wen Zhi Hu participated in mouse exercise training, glucose tolerance test and insulin tolerance test. YuXin Ruan and Hong Ying Chen participated in some of the Western blot experiments. Qiang Zhang analyzed some of the data. Zhe Zhang and Zhe Shu Ding commented on and revised the manuscript and kept a tight check on the whole project. All authors read and approved the final manuscript.

## FUNDING INFORMATION

This study was supported by the National Natural Science Foundation of China (32000836, 31671241), Fundamental Research Funds for the Central Universities (Ministry of Education of the People's Republic of China, 2018ECNU‐HLYT048), and the Construction Project of Key Laboratory of Adolescent Health Assessment and Exercise Intervention of Ministry of Education, East China Normal University (Ministry of Education of the People's Republic of China, 40500‐541235‐14203).

## CONFLICT OF INTEREST STATEMENT

The authors declare that there is no competing interest for this work.

## Data Availability

Data and publication materials are available from the corresponding author on a reasonable request.
